# Females May Have Less Severe Acne, but They Suffer More: A Prospective Cross-Sectional Study on Psychosocial Consequences in 104 Consecutive Polish Acne Patients

**DOI:** 10.3390/jcm13010004

**Published:** 2023-12-19

**Authors:** Marta Szepietowska, Aleksandra A. Stefaniak, Piotr K. Krajewski, Lukasz Matusiak

**Affiliations:** Department of Dermatology, Venereology and Allergology, Wroclaw Medical University, 50-368 Wroclaw, Poland; marta.szepietowska@student.umw.edu.pl (M.S.); aleksandra.stefaniak@umw.edu.pl (A.A.S.); luke71@interia.pl (L.M.)

**Keywords:** acne, gender, quality of life, stigmatization, depression, psychodermatology

## Abstract

Acne is a common skin condition affecting both adolescents and adults, and it can profoundly impact patients’ quality of life and mental well-being. This prospective cross-sectional study aims to explore the differences in psychosocial aspects between male and female acne patients in Poland. A total of 104 consecutive acne patients were included in this study. Clinical severity, patients’ quality of life, stigmatization levels, and psychiatric disturbances were evaluated using the following instruments: Investigator Global Assessment (IGA), Dermatology Life Quality Index (DLQI), Cardiff Acne Disability Index (CADI), 6-Item Stigmatization Scale (6-ISS), and Hospital Anxiety and Depression Scale (HADS). This study found that female patients exhibited significantly less severe acne and experienced significantly decreased quality of life and increased levels of stigmatization. Furthermore, anxiety levels among female patients exceeded those observed in their male counterparts. Notably, no disparities in the severity of depression were observed between the two gender groups. Correlations were discerned among all psychosocial parameters in the entire study cohort and in the female subgroup, while such correlations were not uniformly observed among male participants. This study underscores the importance of considering psychosocial aspects and implementing routine measurements in the management of acne to improve patients’ well-being.

## 1. Introduction

Acne is the most common skin condition. It affects both adolescents and adults [1,2]. Epidemiological studies suggest that about 80% of adolescents and young adults develop acne lesions. In adults, acne affects about 40% of this population, and this type of acne is called “adult acne” or “acne tarda”. The majority of acne subjects present with mild disease intensity; it is estimated that in about 20% of acne patients, the disease is of moderate to severe intensity [3,4]. The most common location of acne is the face; however, the lesions frequently appear on the chest and upper back [1]. Although acne is not a life-treating condition, independently of its clinical severity, it heavily influences patients’ psychosocial status [5,6,7,8,9]. Acne patients have been demonstrated to have significantly lower quality of life (QoL) [9,10] and a higher level of stigmatization [9,11]. Moreover, a European project on dermatology outpatients carried out in 13 countries by the European Society for Dermatology and Psychiatry (ESDaP) showed that acne patients are at increased risk for the development of depression and anxiety reactions. Additionally, suicidality is increased in acne subjects in comparison to healthy controls [12]. However, data on differences in the influence of acne on psychosocial parameters between female and male patients is frightening and ambiguous.

Therefore, in the current study, we decided to analyze the psychosocial status of consecutive acne patients, paying special attention to gender differences.

## 2. Materials and Methods

### 2.1. Patients

This prospective cross-sectional study was undertaken on a group of 104 consecutive patients admitted to the selected dermatology outpatient departments due to their acne problems. Acne was diagnosed by experienced dermatologists based on typical clinical manifestations. Those with additional dermatoses were excluded from this project. The group consisted of 69 (66.3%) females and 35 (33.7%) males at a mean age of 20.38 ± 5.78 years (age range: 14–37 years). They all had acne lasting, on average, 5.97 ± 4.73 years (range: 6 months–20 years). Females reported significantly longer (*p* = 0.048) duration of their acne—6.71 ± 5.06 years (range: 6 months–20 years) than male subjects—4.47 ± 3.61 years (range: 1–15 years). The vast majority of acne patients—72 subjects (69.2%)—had family acne histories. Detailed demographic data of the studied patients, with special attention to gender, is grouped in Table 1.

This study was conducted in Wroclaw, Poland, according to the Declaration of Helsinki rules and achieved the approval of the Ethics Committee of Wroclaw Medical University (KB-250/2023).

### 2.2. Assessments

All patients were diagnosed with acne vulgaris based on clinical presentation by one of the experienced dermatologists participating in the current project. The clinical severity of acne was assessed with the Investigator Global Assessment (IGA), specially designed and validated for acne subjects. The IGA for acne is an instrument that allows physicians to grade patients’ acne on a 5-point scale. Clear skin scored 0 points, and severe acne scored 4 points [13].

Two instruments were used for the assessment of QoL in the studied subjects: the Dermatology Life Quality Index (DLQI) and the Cardiff Acne Disability Index (CADI) [14,15,16]. DLQI is a dermatology-specific questionnaire created specifically to measure QoL impairment in patients suffering from skin diseases. It has a recall period of one week and consists of 10 items. All those items are scored by the patient, giving 0 points to the answer “not at all” and 3 points to the answer “very much”. The final DLQI score ranges from 0 to 30 points. The higher the score, the more decreased QoL is [15]. To assess the influence of the disease on patients’ lives, the following cut-off points were used: 0–1 point—no effect at all; 2–5 points—small effect; 6–10 points—moderate effect; 11–20 points—very large effect; and 21–30 points—extremely large effect [16]. CADI is a disease-specific questionnaire developed especially for acne patients. The recall period is one month, and the instrument has 5 questions scored by the acne subject from 0 points to 3 points. As in DLQI, the higher final score of CADI (sum of all points for 5 questions) indicates a more impaired QoL [14,17]. 

The 6-Item Stigmatization Scale (6-ISS) was employed to measure the level of stigmatization. This is a self-filled questionnaire that contains six questions. Each question is scored from 0 points (not at all) to 3 points (always). The 6-ISS final score ranged between 0 points and 18 points; a higher score indicated a higher level of stigmatization [18,19,20].

Secondary psychiatric disturbances, like depression and anxiety, were assessed with the Hospital Depression and Anxiety Scale (HADS). It is a widely used instrument, containing seven questions related to depression and seven questions related to anxiety. Each question is scored from 0 points to 3 points, giving the final score of each domain ranging from 0 points to 21 points [21,22].

### 2.3. Statistical Analysis

Statistical analysis was performed with IBM SPSS Statistics v. 26 (SPSS INC., Chicago, IL, USA) software. All the data were assessed for parametric and non-parametric distributions using the Shapiro–Wilk normality test. The minimum, maximum, mean, standard deviations, and ranges were given. The Student’s T test or Mann–Whitney U test for parametric and non-parametric data were employed for quantitative data. Depending on normality, Spearman’s and Pearson’s correlations were used for the correlation assessments. Qualitative data were analyzed using the Chi2 test. *p*-values less than 0.05 were considered statistically significant.

## 3. Results

The mean acne severity, assessed with IGA, in the whole study group was 3.22 ± 0.7 points. Female patients presented with significantly (*p* = 0.012) less severe acne than male subjects (3.10 ± 0.71 points and 3.46 ± 0.61 points, respectively). Analyzing various acne severities, a statistically significant difference (*p* = 0.029) was found between both gender groups. Severe and very severe acne was found only in 30.4% of females, whereas 48.6% of males were diagnosed with these grades of acne severity. In contrast, mild acne was found in 20.3% of females and only in 8.6% of males (Table 2).

**Figure 1:**
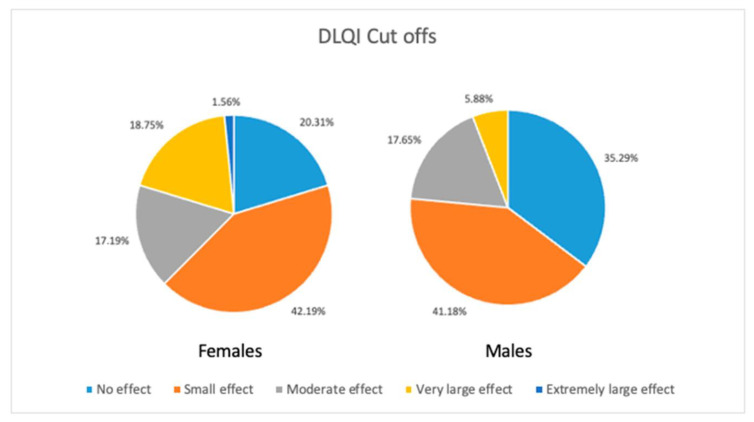
Differences in DLQI cut-offs between sexes.

QoL assessment with an acne disease-specific questionnaire CADI clearly showed that females had significantly (*p* < 0.001) decreased their QoL compared to males (7.48 ± 3.76 points and 4.31 ± 3.21 points, respectively). The numerically decreased QoL in females in comparison to males was also noted when evaluated with the dermatology-specific instrument DLQI (5.86 ± 5.37 points and 3.82 ± 3.82 points, respectively); however, the observed difference did not reach statistical significance. Taking into consideration the cut-off points of DLQI, there was no difference between gender groups (Figure 1).

Our female acne patients appeared to be significantly (*p* = 0.034) more stigmatized than male ones. The mean value of 6-ISS in females was 3.75 ± 2.87 points, and in males, it was 2.62 ± 2.5 points. Additionally, females presented with significantly (*p* = 0.011) higher anxiety levels than males (8.07 ± 4.14 points and 6.06 ± 3.73 points, respectively). However, there was no difference in severity of depression between both analyzed groups (females: 3.93 ± 3.18 points and males: 3.82 ± 2.67 points) (Table 3).

In the whole of our acne patients, as well as in gender groups separately, we were not able to find any significant correlation between the studied psychosocial parameters (CADI, DLQI, 6-ISS, HADS-A, and HADS-D) and the duration of acne. Approximately 6-ISS significantly correlated (r = 0.250, *p* = 0.014) with disease severity. The other psychosocial parameters did not significantly correlate with IGA. The same phenomena were observed separately for females (r = 0.263, *p* = 0.036) and males (r = 0.437, *p* = 0.011). Among all assessed psychosocial parameters in all studied patients, each one correlated with the other (Table 4).

This was also true for the group of females. However, among males, no significant correlations were found between CADI and HADS-A, CADI and HADS-D, or between 6-ISS and HADS-D (r = 0.226; *p* = 0.129; r = 0.227; *p* = 0.196; and r = 0.321; *p* = 0.06, respectively).

## 4. Discussion

Acne is a chronic inflammatory disease of prolonged course with recurrences and relapses. It affects about 650 million people worldwide, making it the 8th most common disorder globally [23,24]. Acne is regarded as one of the most common reasons for visiting the [25] doctor. The disease has a profound negative influence on patients’ well-being [5,6,7,8,9,10,11,12]. Therefore, the European Dermatology Forum S3-Guideline for the Treatment of Acne strongly recommends consideration of health-related QoL in acne patients as routine daily measurement as well as the assessment of treatment effectiveness [26]. 

There is no doubt that the OoL of acne patients has markedly decreased. This was also confirmed in the current study, which showed small to moderate QoL impairment in the studied group of acne subjects. This data are in agreement with previous studies documenting the DLQI scores in acne patients between 2.8 and 6.42 points [8,13,15,27,28]. We are aware of only one study, concentrated exclusively on patients with severe acne, whose DLQI mean score was above 10 points [29]. Analyzing gender differences and QoL impairment in acne patients, we noticed that females had a decreased QoL in comparison to males. This was visible for both our assessments: CADI and DLQI; however, the difference was significant only when the QoL was evaluated with CADI. In our previous cross-sectional study conducted on a group of acne university students (mainly with minimal and mild acne lesions), we found that DLQI scores were significantly higher in females than in males (3.17 ± 3.74 and 1.76 ± 2.69 points, respectively) [8]. Also, other authors reported more decreased QoL in their female acne patients than acne male subjects [30], but there are also some reports showing no difference in QoL impairment among acne patients between both genders [31]. The previous experience of our group analyzing QoL in several different dermatoses clearly confirmed that in hidradenitis suppurativa and dermatophytosis patients, females demonstrate significantly lower QoL in comparison to male patients suffering from similar disease activity [32]. Moreover, in other dermatoses, like psoriasis, atopic dermatitis, or vitiligo, females tended to suffer more than males [33,34,35]. 

Stigmatization is defined as a discrediting mark, biological or social, that sets a person apart from others and disrupts interactions with them [36]. It is a common phenomenon among patients suffering from skin diseases. However, the literature on feeling stigma in different dermatoses is rather scarce, especially if compared to the very numerous papers dealing with QoL in dermatology patients [36]. Based on a recent review, one may conclude that stigmatization has been studied up until now only in 20 different dermatoses, including acne. Acne stigmatization papers constituted only 5% of all analyzed papers [36]. Recently, we conducted a study on stigmatization among acne patients among a group of university students. Screening more than 700 students, we found stigmatization significantly more common in subjects with acne in comparison to non-acne subjects. Moreover, the level of stigmatization, assessed with 6-ISS as 1.68 ± 2.42, was significantly higher in students with acne [9]. It is worth mentioning that this study was population-based, and the vast majority of students (around 90%) presented with acne of minimal and mild severity [9]. In the current cohort of consecutive acne patients, the level of stigmatization was higher and assessed at 3.36 ± 2.79 points according to 6-ISS. We clearly showed that female acne patients were more stigmatized than male acne subjects. A similar observation was documented in the previously mentioned study among acne university students. Acne is a visible disease with predominantly facial location; females in general pay more attention to esthetic aspects than males [37]. It is of interest that acne severity was not different between female students and male students with acne [9]. To the best of our knowledge, these are the only two available papers analyzing stigmatization in different gender groups of acne patients. Previous studies conducted by our group in patients with psoriasis and hidradenitis suppurativa showed no difference in the level of feeling stigma between females and males [38,39]. This may illustrate that acne, with its lesions predominantly located on visible skin areas, mainly the face, is a very specific entity. It was suggested that females may experience a higher level of stigmatization as, in general, aesthetic aspects of the skin are more important for them than for males [37]. Interestingly, in a recent European project, the perceived stigmatization was significantly higher in all dermatology patients, including acne sufferers, than in healthy controls [40]. However, male sex, together with lower age, being single, and higher disease severity, appeared to be the predictive factors for stigmatization [40]. In our cohort of acne patients, we also found a significant correlation between the level of stigmatization and acne clinical severity. Exactly the same finding was documented in university students with acne [9]. 

Acne affects individuals not only physically but also psychologically [41]. Several authors have documented that psychiatric comorbidities like depression, anxiety, and suicidality are increased in acne subjects, especially in adolescent populations [12,41]. Our results confirmed these findings and suggest that female acne patients present with markedly higher anxiety than males. This is in agreement with a previous study showing that the female sex had an increased likelihood of having abnormal anxiety (OR 2.451 [95% CI: 1.072–5.607]) [41]. Interesting observations are coming from a very recent European project on body dysmorphic disorder in patients with various dermatoses [42]. Although there was a highly significant correlation between depression, anxiety, and stigmatization in patients with skin problems, stigmatization appeared to be a more relevant predictor of body dysmorphic disorder than depression, anxiety, and suicidal ideations [42]. This can clearly suggest the necessity of introducing anti-stigma strategies to dermatological patients as early as possible in order to reduce the risk of developing clinically relevant body dysmorphic disorder. This is especially true for acne patients. Body dysmorphic disease was found in almost 17% of acne patients, and the risk for this psychiatric disturbance was seven times higher as compared to healthy individuals. It is worth mentioning that the occurrence of body dysmorphic disorder was significantly related to younger age, higher self-rated stress, and female gender [42]. 

We are aware of some limitations of the current study. The patients for this project were recruited only in one center, and they were coming from one geographical and cultural region. It will be of interest to conduct an international multicenter study paying special attention to the differences in psychosocial status of acne patients of different genders. One could not completely exclude cultural factors playing an important role in visible dermatosis, as acne is. Additionally, the multicenter study will allow us to recruit more patients, making the final results more representative of the whole acne population. The lack of a healthy control group is another important limitation of the project. It is worth mentioning that the consecutive sampling typology, having a 2:1 F:M ratio, could have impacted the differences that emerged. It is important to notice that the assessment of psychiatric comorbidities, such as depression and anxiety, was based here on only one instrument—HADS. Although HADS is a well-known and widely used questionnaire, the employment of additional instruments such as the Patient Health Questionnaire-9 (PHQ-9) [43] and the Generalized Anxiety Disorder-7 (GAD-7) [44] will definitely add value.

## 5. Conclusions

Female acne patients, independently of the disease severity, exhibit more severe impairment in quality of life, higher stigmatization levels, and greater anxiety compared to males. These observations are of importance in a holistic approach to acne patients, suggesting the role of psychological consulting in at least some acne subjects.

## Figures and Tables

**Table 1:** Patients’ demographics.

Characteristic	Whole Group	Women	Men	P
Sex, n (%)	104 (100)	69 (66.3)	35 (33.7)	NA
Acne duration (years) mean ± SD	5.97 ± 4.73	6.71 ± 5.06	4.47 ± 3.61	0.048
Familiarity, n (%)	72 (69.2)	49 (71)	23 (65.7)	NS

n—number of subjects; SD—standard deviation; NA—not applicable; NS—non-significant.

**Table 2:** Acne severity in the studied population was assessed with IGA and CADI.

Acne Severity	Whole Group (n = 104)	Females (n = 69)	Males (n = 35)	Females vs. Males P
IGA, mean ± SD	3.22 ± 0.7	3.10 ± 0.71	3.46 ± 0.61	0.012 0.029
1 Mild	17 (16.4)	14 (20.3)	3 (8.6)	
2 Moderate	49 (47.1)	34 (49.3)	15 (42.9)	
3 Severe	28 (26.9)	18 (26.1)	10 (28.6)	
4 Very severe	10 (9.6)	3 (4.3)	7 (20.0)	

n—number of subjects; SD—standard deviation; IGA—Investigator Global Assessment; CADI—Cardiff Acne Disability Index.

**Table 3:** Psychosocial assessment in the studied population.

Characteristic	All Group (n = 104)	Females (n = 69)	Males (n = 35)	P
CADI, mean ± SD	6.41 ± 3.87	7.48 ± 3.76	4.31 ± 3.21	<0.001
HADS A, mean ± SD	7.41 ± 4.1	8.07 ± 4.14	6.06 ± 3.73	0.011
HADS D, mean ± SD	3.89 ± 3.01	3.93 ± 3.18	3.82 ± 2.67	NS
6-ISS Sum, mean ± SD	3.36 ± 2.79	3.75 ± 2.87	2.62 ± 2.5	0.034

n—number of subjects; CADI—Cardiff Acne Disability Index; SD—standard deviation; NS—non-significant; 6-ISS—6 Item Stigmatization Scale; HADS—Hospital Anxiety and Depression Scale; A—Anxiety; D—Depression.

**Table 4:** Correlations between psychosocial assessments.

	CADI	DLQI	HADS A	HADS D	6-ISS
CADI	NA	r = 0.807 *p* < 0.001	r = 0.576 *p* < 0.001	r = 0.553 *p* < 0.001	r = 0.603 *p* < 0.001
DLQI	r = 0.807 *p* < 0.001	NA	r = 0.454 *p* < 0.001	r = 0.483 *p* < 0.001	r = 0.615 *p* < 0.001
HADS A	r = 0.576 *p* < 0.001	r = 0.454 *p* < 0.001	NA	r = 0.724 *p* < 0.001	r = 0.503 *p* < 0.001
HADS D	r = 0.553 *p* < 0.001	r = 0.483 *p* < 0.001	r = 0.724 *p* < 0.001	NA	r = 0.457 *p* < 0.001
6-ISS	r = 0.603 *p* < 0.001	r = 0.615 *p* < 0.001	r = 0.503 *p* < 0.001	r = 0.457 *p* < 0.001	NA

CADI—Cardiff Acne Disability Index; DLQI—Dermatology Life Quality Index; 6-ISS—6-Item Stigmatization Scale; HADS—Hospital Anxiety and Depression Scale; A—Anxiety; D—Depression; r—Spearman coefficient; NA—not applicable.

## Data Availability

Data are available upon reasonable request from the corresponding author.
